# Prevalence of Metabolic Syndrome According to Physical Activity, Dietary Habits, Mental Status, Social Status, Health Behavior, and Obesity Phenotypes in Korean Adolescents: 2016–2021

**DOI:** 10.3390/foods12173304

**Published:** 2023-09-02

**Authors:** Xiangxiang Dou, Yonghwan Kim, Hyunsik Chu

**Affiliations:** 1 Department of Sports Welfare Education, Woosuk University, Wanju 55338, Republic of Korea; douxiang@stu.woosuk.ac.kr; 2 Department of Physical Education, Gangneung-Wonju National University, Gangneung 25457, Republic of Korea; 3 Department of Forest Leisure Sprots, Gangneung Yeoungdong University, Gangneung 25521, Republic of Korea

**Keywords:** adolescent, metabolic syndrome, aerobic, breakfast, depression, stress, household income, dietary habits

## Abstract

Environmental factors play a role in increasing or decreasing the risk of metabolic syndrome (MetS) in adolescents. We analyzed the impact of physical activity (PA), dietary habits, and mental and socioeconomic status on MetS prevalence in 2143 (boys: 1113, girls: 1030, age: 13–18 years) Korean middle- and high-school students. Metabolically healthy obesity and metabolically unhealthy normal weight were also evaluated. MetS occurred in 215 participants (10.0%), and boys had a higher MetS rate than girls. There was no significant difference in alcohol consumption and smoking experience between individuals with and those without MetS. The odds ratio (OR) for high-school students was 1.33 (95%CI, 1.001–1.789, *p* = 0.043) times that of middle-school students. Depression, low aerobic PA, and high sedentary time increased the ORs to 1.64 (95%CI, 1.059–2.539, *p* = 0.020), 1.52 (95%CI, 1.092–2.203, *p* = 0.003), and 1.86 (95%CI, 1.342–2.587, *p* < 0.001), respectively. Higher energy intake and low weekly breakfast consumption frequency yielded ORs of 1.46 (95%CI, 1.046–2.555, *p* = 0.025) and 1.70 (95%CI, 1.244–2.339, *p* = 0.011), respectively. Strength training, stress, suicidal ideation, dining out frequency, and household income did not impact MetS prevalence. Despite obesity, MetS decreased by 29.7% with high aerobic PA and 37.9% with high weekly breakfast consumption frequency. In conclusion, MetS risk was higher for men, individuals with depression, and high-school students. Low aerobic activity, high calorie intake, and low weekly breakfast consumption frequency increased MetS risk. Despite obesity, high aerobic activity, low sedentary time, and breakfast consumption was associated with lower MetS risk.

## 1. Introduction

Traditionally, metabolic syndrome (MetS) and cardiovascular diseases were considered exclusive to adults; however, currently, the prevalence of MetS in adolescents is reportedly in the range of 0.3–26.4% [1]. The number of obese adolescents is increasing in many countries owing to high-calorie diets and low physical activity (PA); consequently, the occurrence of MetS is also increasing [2]. Health experts have consistently warned about an increased risk of obesity among adolescents. A study of adolescent obesity rate in the United States revealed an increase from 13.9% in 2000 to 18.5% in 2016 [3], whereas China exhibited an increase from 6.3% in 2000 to 10.2% in 2015 [4]. Adolescent obesity is a serious issue, because obesity in 80% of the individuals continues into adulthood, with 70% remaining obese after the age of 30 years [5].

MetS is diagnosed when typical cardiovascular risk factors such as abdominal obesity, high blood pressure (BP), and high glucose, high triglyceride (TG), and low high-density lipoprotein cholesterol (HDLC) levels exist concomitantly [6]. MetS requires early management because it is a potential risk factor for long-term myocardial infarction, stroke, and angina pectoris. In a previous study, patients with MetS showed a 1.46-fold increase in death due to myocardial infarction and a 1.43-fold increase in the risk of stroke compared to patients without MetS [7].

The exact cause of MetS in adolescents has not been fully elucidated; however, it is believed to be related to a combination of genetic and environmental factors. Studies in adults indicated that high PA and a balanced diet will help prevent and address MetS [8,9]. However, another study found no relationship between PA and MetS in adolescents [10]. This is because of the complexities involved in the studies conducted in adolescents. Adolescents live in a unique environment that differs from that of adults, as they spend most of their time in the limited space of systematically planned schools and homes. Therefore, they are impacted by factors such as school culture, school lunch services, and physical education classes [11].

Stress and depression have been shown to increase the prevalence of MetS; however, the direct mechanism underlying the development of MetS has not been clearly identified [12]. Stress negatively affects appetite, fat and carbohydrate metabolism, and insulin activity, and can cause a decrease in PA [13]. Men and women with high work stress exhibit two-fold and five-fold increased risks of developing MetS, respectively [14]. The causes of stress in adolescents vary. Attending school, friendships, academic stress, puberty, and mental status factors such as relationships with parents and family circumstances are linked [15]. Although previous MetS studies in adolescents have focused on PA and dietary habits, few studies have examined the mental health of adolescents. Furthermore, because of the lower prevalence of MetS in adolescents compared to that in adults, the sample size is often relatively small, and studies comprehensively addressing physical and mental status, dietary habits, and household income are rare.

Therefore, we analyzed the mental status, PA, and dietary habits of a relatively large adolescent cohort. Further, metabolically healthy obesity (MHO) and metabolically unhealthy normal weight (MUNW) have rarely been studied. In this study, the characteristics of MHO, which did not correspond to MetS, despite obesity, and those of MUNW, in which MetS occurred despite normal weight, were analyzed to provide evidence for preventive measures against MetS. We hypothesized that physical, mental, and health habits in adolescents would affect MetS and that different characteristics would be displayed according to obesity phenotypes.

## 2. Materials and Methods

### 2.1. Research Process and Participants

**Figure 1:**
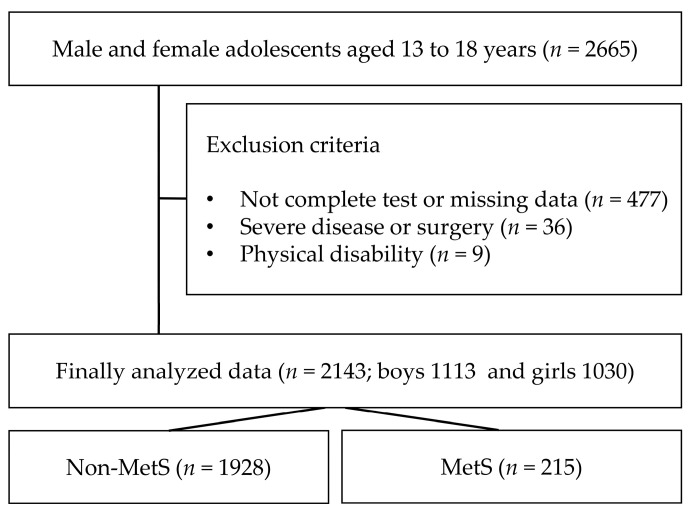
Flowchart of study participants.

The Korea National Health and Nutrition Examination Survey is an annual health project conducted by the Centers for Disease Control and Prevention in Korea that includes all age groups across the country. Participation in the study was voluntary, and only data from those who consented to participate in testing, data analysis, and publication for research purposes were included. Exclusion conditions include people with a history of congenital disease, type 1 diabetes, cancer, or physical disability. In this study, 2143 middle- and high-school students (boys: 1113; girls: 1030) aged 13–18 years old surveyed from 2016 to 2021 were selected (Figure 1). Only data with completed questionnaires (PA, dietary habits, mental status, household income, smoking, and alcohol consumption), MetS, and obesity factors were analyzed. The participants were asked to complete the questionnaire, and the staff only provided assistance if the participant had difficulty understanding the sentence or had poor eyesight.

Participants were instructed to fast for 8 h before the test; light gowns and slippers were provided. The examination procedure was completed after consultation with a physician, obtaining written consent, and completing the questionnaires. Body and BP measurements and blood sampling were performed. Since the participants were minors, the staff provided written and verbal explanations and obtained written consent from their legal guardians. This study complied with the guidelines of the Declaration of Helsinki and was approved and supervised by the Research Ethics Committee of Gangneung-Wonju National University (2020-16).

### 2.2. Questionnaire: Leisure PA, Dietary Habits, Alcohol, Smoking, Household Income

PA: The PA questionnaire was based on the International PA Questionnaire (IPAQ) developed and disseminated by the World Health Organization (WHO) [16]. In this study, aerobic PA and strength training frequency were selected among PA [17]. The PA survey used the 7-day memory recall method, and participants were asked to complete the questionnaire independently. PA was classified as aerobic PA and strength training and was divided into high-, medium-, and low-intensity PA. The participation date and times per week were recorded. Average sedentary time per day was also determined. Sedentary time refers to inactivity spent sitting, excluding sleep. It includes quiet time, such as studying, playing games, watching TV, using the computer, or listening to music. The variables were classified as low, medium, and high. The level of aerobic during PA followed the guidelines of the American College of Sports Medicine [18].

Dietary habits: A dietary habits questionnaire developed by the survey host was used, and its reliability and validity were tested [19]. The items evaluated in this study were extracted and analyzed. Daily total calories, weekly frequency of dining out, weekly breakfast consumption frequency, and experience of receiving nutrition education were investigated. Calorie classification was denoted as low for the recommended calorie intake by age, medium for the standard intake, and high for the intake of more than the recommended calorie intake by age [20]. Energy intake was investigated using the 24-h recall method. For the food frequency questionnaire, a questionnaire developed for the Korea National Health and Nutrition Examination Survey was used [21]. This food frequency questionnaire consisted of 112 food or food items, and the frequency or amount of intake was investigated based on the daily recommended nutrient intake for each age group [22]. Therefore, the method of calculating the nutritional components and calories included in the food composition table was used.

Eating out frequency was classified as low for ≤ 4 times a week, medium for 5–6 times a week, and high for 1–2 times a day. Weekly breakfast consumption frequency was categorized as high for ≥5 days, medium for 3–4 days, and low for ≤2 days [23,24]. Furthermore, we surveyed the participants regarding their experience of receiving nutrition education.

Mental status: The questionnaire about stress and depression used in the survey was created based on the Patient Health Questionnaire-9 (PHQ-9), and mental status for the previous 2 weeks was investigated [25]. Additionally, the participants were asked whether they had contemplated suicide in the past year.

Household income, as determined by parents, is a representative factor in determining socioeconomic status. A quintile set by the country based on monthly household income was used. Among them, the middle group was analyzed as medium-income, the lower group as low-income, and the upper group as high-income.

Alcohol and smoking: We investigated smoking experience, first smoking age, smoking amount, and frequency of smoking. Additionally, alcohol experience, first alcohol experience age, type of alcohol consumed, and frequency of alcohol consumption were included in the questionnaire. In this study, participants’ experiences were included in the analysis [26].

### 2.3. Body Measurements, Blood Collection, and BP

An electronic automatic height gauge and weight scale were used for body measurements. Body mass index (BMI) was calculated using the measured height and weight. Waist circumference was measured horizontally at the thickest part near the belly button using a tape measure. If an error of 0.5 cm or more occurred after measuring twice, it was measured again, and the highest value was used. For blood collection, participants fasted for 8 h, and a skilled nurse collected blood samples from the median cubital vein. A compression band was applied on the upper arm to enhance vein visibility and the vein was lightly tapped with the second and third fingers. A needle was inserted into the vein at an angle of 15–30°, and a total of 25–30 mL of blood was collected using a vacuum tube. Blood analysis was performed using an automatic blood analyzer (Hitachi-747, Tokyo, Japan). The TG, glucose, and HDLC levels were analyzed from the blood samples. BP was measured three times using an electronic BP monitor, and the average values of the second and third measurements were used for analysis.

### 2.4. Diagnosis MetS and Obesity

The diagnosis of MetS in adolescents was based on the criteria proposed by Cook et al. [27]. Obesity factors and BP were determined using the 90th percentile based on previous Korean studies [28,29]. Waist circumference (WC): WC was considered abnormal if it corresponded to the 90th percentile according to age and sex [28] or if it was ≥90 cm in men and ≥85 cm in women by applying the adult standard. BP: BP was considered elevated when systolic blood pressure (SBP) and/or diastolic blood pressure (DBP) exceeded the 90th percentile for age, sex, and height in adolescents [29] or if it was >130/85 mmHg, which is equivalent to the adult standard; participants were classified into blood pressure groups. Glucose: Hyperglycemia is defined as fasting blood glucose > 100 mg/dL. TG: TG levels > 110 mg/dL were considered high. HDLC: Low HDLC levels were <40 mg/dL in men and <50 mg/dL in women. In addition, a drug history related to MetS was classified as a diagnosis. MetS was diagnosed when three or more of the five diagnostic criteria were met. 

Obesity was classified using BMI, and it was classified as above the 90th percentile according to age and sex in Korea [28] or above BMI 25 kg/m^2^ according to the Asia-Pacific criteria of the WHO [30].

### 2.5. Data Analysis

Data were analyzed using SPSS software (version 25.0 (SPSS Inc., Chicago, IL, USA)). The Shapiro–Wilk test was performed for normality, and the MetS factors, which are the main variables analyzed in this study, exhibited normal distribution. Continuous variables are expressed as means and standard deviations, and parametric statistical methods were applied. An independent *t*-test was used for between-group comparisons, and the chi-square test was used for categorical variables. For MetS prevalence, logistic regression analysis was performed to calculate the odds ratio (OR), and adjustment variables included sex, school, depression, aerobic PA or sitting time, and energy intake or breakfast, which were significant in the odds ratio analysis. Linear regression analysis and step selection were performed using only the variables significant in the OR to confirm the entry and exclusion variables, and a model was used to confirm the order of the entry variables. The significance level for all analyses was set at *p* < 0.05, and the confidence interval (CI) of the odds was set at 95%.

## 3. Results

### 3.1. General Characteristics and Sociological Status of Participants with and without MetS

MetS occurred in 215 participants (10.0%). Compared to the non-MetS group, the MetS group exhibited significant differences in height, weight, and BMI, as well as MetS factors such as WC, BP, TG, and glucose. The prevalence rates of obesity were 14.6% and 75.8% in the non-MetS and MetS groups, respectively. There were no significant differences between the groups in terms of age, household income, alcohol consumption, or smoking history. PA participation ≥ 3 times per week was also higher in the non-MetS group than in the MetS group (Table 1). Additionally, obesity and normal weight were classified based on BMI; MHO and MUNW, which are the main analysis variables in this study, were 14.6% and 24.2%, respectively.

### 3.2. MetS OR According to Social and Mental Status Health

The prevalence of MetS was 11.5% for boys and 8.4% for girls, with a significantly higher occurrence in boys. There were no significant differences in the OR of MetS based on household income, stress factors, or suicidal ideation. High-school students had higher MetS prevalence than middle-school students, and the OR increased 1.3 times. Adolescents who experienced depression during the previous 2 weeks had a 1.6-fold higher MetS OR than those without depression (Table 2).

### 3.3. MetS OR According to PA and Dietary Habits

Dietary habits and PA are representative MetS management methods, along with obesity management. Table 3 shows the prevalence of MetS according to the PA status and dietary habits. The prevalence of MetS in the low aerobic PA group had an increased OR of 1.5 times compared to the the high aerobic PA group but was not significant for strength training. However, long sedentary time, indicating inactivity, increased the risk of MetS 1.8-fold. In addition, in terms of energy intake, the OR for MetS increased 1.46-fold in those who ate excessively beyond the recommended calorie standards. In addition, the OR of MetS increased by 1.70-fold in the group that had a low weekly breakfast consumption frequency compared to the group with a high weekly breakfast consumption frequency. In this study, there was no significant difference regarding dining out frequency or MetS prevalence according to nutrition education level.

### 3.4. Relationship between PA and Dietary Habits in MHO and MUNW

Based on the results showing significance in Table 2 and Table 3, a multiple stepwise regression analysis was performed to identify the factors that exerted greater influence on MetS. In the results, sedentary time, breakfast, energy intake, sex, and aerobic PA became entry variables, and we used these as adjustment variables. 

**Figure 2:**
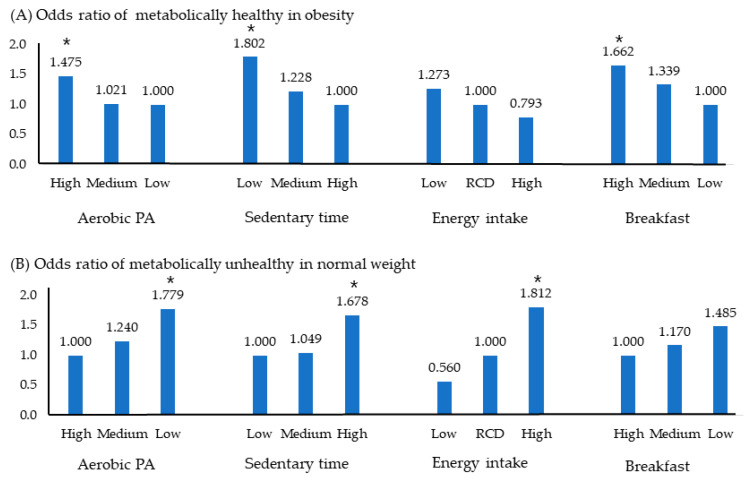
Odds ratio of MHO and MUNW according to health related behavior. Significance level, * *p* < 0.05; Logistic regression analysis was used. Abbreviations: MetS, metabolic syndrome; OR, odds ratio; PA, physical activity; RCD, recommended amount; MHO, metabolically healthy obesity; MUNW, metabolically unhealthy normal weight.

Figure 2 shows the OR of MHO and MUNW using the independent variables selected in Table 3. Figure 2A represents the OR of MHO for adolescents who were metabolically healthy despite being obese. The likelihood of being metabolically healthy increased 1.4-fold with aerobic PA, 1.8-fold with low sedentary time, and 1.6-fold in adolescents with high weekly breakfast consumption frequency.

Figure 2B depicts the OR of MUNW for adolescents who were metabolically unhealthy despite having a normal weight. It increased 1.7 times in the low aerobic PA group, 1.6 times in the long sitting group, and 1.7 times in the high energy intake group. However, breakfast status did not significantly affect prevalence.

## 4. Discussion

The main result of this study is that the prevalence of MetS in adolescents was lower in the group with well-managed health behaviors. Low PA and high sedentary time are associated with increased prevalence of MetS in adolescents [31]. In previous cross-sectional design studies, boys who exercised for less than 60 min a day had a MetS prevalence of 11.4%, approximately twice as high as the 5.3% prevalence observed in the group exercising for more than 60 min [32]. Additionally, a meta-analysis reported a 1.35-fold increased risk of MetS in the group with low-level PA [33]. The significant association between PA and MetS did not differ according to the survey method used. In an analysis using the questionnaire method, MetS risk increased by 1.23 times in low PA group; it increased 2.93 times when PA was measured using an accelerometer [33].

However, there have been studies showing different results. A previous study found that MetS is affected more by longer sedentary time than by PA [10]. In addition, another study found an increased risk of MetS in groups that watched TV and used computers for more than 2 h on weekends [33]. In our PA-related analysis, strength training showed no significant results. Many studies have reported that both aerobic PA and strength training effectively improve MetS factors and that the low-strength group exhibits an increased prevalence of MetS [34,35].

Nonetheless, in a study investigating arteriosclerosis according to PA habits, similar to the results of the present study, it was reported that only aerobic activity was significant, and there was no significant difference regarding strength training [36]. Researchers also reported that <1 h of strength training per week could reduce the risk of MetS by 25% [37]. Nevertheless, sports medicine specialists recommend a combination of aerobic and strength training. Glucose metabolism occurs in the muscles, and increasing muscle mass lowers insulin resistance and promotes gluconeogenesis [34,38]. In addition, one of the analyses emphasized in this study was the analysis of the factors of MHO. In this study, despite obesity, increasing aerobic PA and reducing sedentary time played a role in reducing MetS; these results are similar to those of a previous study [39].

An interesting finding in the current study was that poor breakfast intake increased the prevalence of MetS. These results show the same trend as those reported in previous studies. In a study conducted on 889 adults, the OR for MetS increased 1.68-fold when individuals at the age of 43 years ate a poor breakfast [40]. Another long-term study lasting approximately 20 years, including childhood and adulthood, revealed that breakfast skippers exhibited significantly higher WC and fasting insulin levels, as well as higher low-density lipoprotein cholesterol and total cholesterol levels [41].

Nutrition and health experts continue to emphasize the importance of breakfast, but the underlying mechanism is still under investigation. The likely mechanisms mentioned so far are as follows. Skipping breakfast can lead to high insulin resistance. In a study investigating the dietary habits of elementary school students, it was reported that breakfast consumption showed an inverse correlation with the insulin resistance index (HOMA-IR) [42], which not only improves overall meal quality but also plays a role in lowering the risk of insulin resistance [43]. Furthermore, skipping breakfast increases stress hormones, such as cortisol, increases glucose levels, and promotes the accumulation of abdominal fat. An examination of daily meal patterns and cortisol levels in women revealed that breakfast skippers showed higher cortisol levels by the midafternoon than those who consumed breakfast [44]. The action of these stress hormones and psychological compensation for skipping breakfast will cause individuals to eat more during lunch or dinner, which will eventually lead to an increase in total calorie consumption [45]. The results of the present study are similar. People with MetS do not eat breakfast and consume more calories than those without MetS. In particular, considering that breakfast had a significant effect on MHO but not on MUNW, the importance of breakfast should be emphasized more in the obese group than in the normal-weight group.

The opinions about breakfast vary. One of the main opinions is that a healthy breakfast can have a positive impact on weight management. A nutritious breakfast can feel full and control cravings, leading to better portion control and food choices, which can prevent overeating later. Conversely, when people skip breakfast, they are more likely to experience extreme hunger later in the day, which can lead to overeating. Also, skipping breakfast can cause blood glucose levels to spike and then plummet, increasing hunger and cravings for unhealthy foods [42]. Nonetheless, it should be noted that the relationship between breakfast and obesity is complex and individual factors play a role. There is an opinion that skipping breakfast has the potential to reduce overall calorie intake. In addition, since the factors related to obesity are very diverse, it is argued that breakfast does not have an absolute effect. A healthy breakfast is generally recommended but not a guaranteed solution to preventing or treating obesity [46].

Eating out did not yield significant results in this study. Along with economic growth, the dining out industry is developing, and eating out is gradually increasing owing to the increase in dual-income couples. However, dining out along with skipping breakfast is considered a negative risk factor for MetS [47]. Therefore, even if not significant, in-depth research should continue along with nutrition education.

Household income was not significantly different in this study. However, many studies have emphasized that parental socioeconomic status influences the prevalence of obesity and MetS [35,48,49]. This is because parents’ high education and economic status naturally provide a good nutritional environment for their children based on high health awareness and a balanced diet [50]. Perhaps the fact that this study shows different results from previous studies raise doubts about the accuracy of the questionnaire for sensitive information, and there is a possibility that participation by the low-income class is low, even in such a survey. In our study, the survey participation rate was higher among high-income households than among low-income households, although the income standard was prepared according to the national classification table.

In addition, a study conducted among adults reported that the risk of MetS increased by 45% in highly stressed individuals, the OR of persons with occupational stress increased by 1.69 times, and suicidal ideation was found to have a significant relationship with diabetes [51,52]. However, mental status and MetS design studies are lacking compared to studies on PA or dietary habits in adolescents and adults.

The pathways and mechanisms by which negative mental status leads to MetS remain unclear. However, this negative mental state could directly affect MetS by increasing the levels of cortisol, a hormone produced by the adrenal glands. High cortisol levels can lead to an increase in glucose levels, a component of MetS, high BP, abdominal fat, and insulin resistance. It is also considered as a contributing factor to MetS because it indirectly leads to unhealthy behaviors, such as overeating, sedentary lifestyle, and lack of sleep [51].

This study had several limitations. Except for the measured values related to MetS, the remaining questionnaires were based on memory. Surveys conducted based on the information from the last week, 2 weeks, and 1 year inevitably display considerable variability. In particular, memory in a state where there are various possibilities for a student, such as vacation or exam period, will tend to overestimate or underestimate uniformity. There are multiple questionnaires that the participants must complete daily. This might result in low concentration and high error potential. Although a large-scale investigation was conducted in this study, the low prevalence remained a limiting factor in the analysis. Therefore, in this study, men and women could not be distinguished and age could not be subdivided. Although the questionnaire on dietary habits was diverse, several items commonly known as healthy dietary habits were extracted and analyzed in this study, and detailed nutritional components were not included in the analysis. One of the major limitations of this study is that it was a cross-sectional study, and it is difficult to explain causality because of successive factors between factors. For example, it is difficult to ascertain whether low PA is due to depression or whether depression is caused by low PA. In the future, more detailed and explanatory research should be conducted to overcome these limitations. Additionally, there are few studies on adolescents, and the challenges they face during their internal and external growth periods differ from those of adults; therefore, extensive research should be continued. In this study, PAs were investigated; however, the actual level of participants’ physical fitness was not investigated. In addition, an analysis considering the environmental characteristics of adolescents will enhance the value of future research. For example, academic status, sleep quality, and home environment, including parental influence, should be considered in future studies.

## 5. Conclusions

The prevalence of MetS was observed to be higher in boys and high-school students than in girls and middle-school students. Low aerobic PA, high calorie intake, low breakfast consumption frequency, and depression increased the risk of MetS. In addition, despite obesity, high aerobic PA, low sedentary time, and high breakfast frequency were metabolically healthy. On the other hand, low PA, high sedentary time, and high energy intake were metabolically unhealthy despite normal weight. Meanwhile, household income, suicidal ideation, stress awareness, strength training, eating out frequency, nutrition education, alcohol, and smoking experience were not significantly associated with MetS.

## Figures and Tables

**Table 1:** General characteristics and sociological status according to MetS status.

Variables	Non-MetS (*n* = 1928)	MetS (*n* = 215)	*t* or x^2^	*p*
Sex, boys/girls	985 (51.1%)/943 (48.9%)	128 (59.5%)/87 (40.5%)	5.527	0.019
Age, years	15.4 ± 1.7	15.5 ± 1.7	−0.943	0.346
Height, cm	166.1 ± 8.1	168.1 ± 8.5	−3.444	<0.001
Weight, kg	58.5 ± 11.9	79.9 ± 14.4	−24.492	<0.001
BMI, kg/m^2^	21.1 ± 3.3	28.1 ± 4.0	−28.740	<0.001
WC, cm	71.3 ± 9.1	89.2 ± 10.3	−27.179	<0.001
SBP, mmHg	108.1 ± 9.5	118.0 ± 11.1	−14.277	<0.001
DBP, mmHg	67.5 ± 8.2	72.9 ± 9.2	−8.970	<0.001
TG, mg/dL	79.9 ± 39.0	148.8 ± 79.0	−21.447	<0.001
HDLC, mg/dL	52.4 ± 9.6	41.3 ± 6.4	16.490	<0.001
Glucose, mg/dL	90.7 ± 7.1	96.9 ± 20.4	−9.346	<0.001
Obesity, *n*	MHO 281 (14.6%)	MUO 163 (75.8%)	441.623	<0.001
Normal weight, *n*	MHNW 1647 (85.4%)	MUNW 52 (24.2%)		
PA attendance, *n*	1925 (72.0%)	134 (62.3%)	8.796	0.003
Household income, KW	560.8 ± 311.2	522.1 ± 304.1	9.881	0.078
Alcohol exp, *n*	585 (30.3%)	68 (31.6%)	0.151	0.698
Smoking exp, *n*	200 (10.4%)	23 (10.7%)	0.022	0.906

Significance level, *p* < 0.05; An independent *t*-test and chi-square test were used. Abbreviations: MetS, metabolic syndrome; BMI, body mass index; BW, body weight; WC, waist circumference; SBP, systolic blood pressure; DBP, diastolic blood pressure; TG, triglycerides; HDLC, high-density lipoprotein cholesterol; PA, physical activity; KW, Korean Won (currency); exp, experience; MHO, metabolically healthy obesity; MUO, metabolically unhealthy obesity; MHNW, metabolically healthy normal weight; MUNW, metabolically unhealthy normal weight.

**Table 2:** MetS odds ratio according to social and mental status health.

Variables	non-MetS (*n* = 1928)	MetS (*n* = 215)	x ^2^	*p*	OR
Sex					
boys (*n* = 1113)	985 (88.5%)	128 (11.5%)	5.572	0.021	Reference
girls (*n* = 1030)	943 (91.6%)	87 (8.4%)			0.728 (0.546–0.972)
Social section					
Household income					
High	1002 (52%)	106 (49.3%)	0.693	0.707	Reference
Medium	450 (23.3%)	55 (25.6%)			1.073 (0.721–1.596)
Low	476 (24.7%)	54 (25.1%)			0.929 (0.658–1.313)
School					
Middle school	997 (51.7%)	95 (44.2%)	4.384	0.036	Reference
High school	931 (48.3%)	120 (55.8%)			1.338 (1.001–1.789)
Mental section					
Depression					
No	1773 (92.0%)	188 (87.4%)	5.082	0.024	Reference
Yes	155 (8.0%)	27 (12.6%)			1.640 (1.059–2.539)
Stress awareness					
None	1407 (73.0%)	149 (69.3%)	1.612	0.447	Reference
A little	454 (23.5%)	59 (27.4%)			0.993 (0.447–2.203)
Max or Many	67 (3.5%)	7 (3.3%)			1.230 (0.893–1.693)
Suicide ideation					
No	1857 (96.3%)	209 (97.2%)	0.444	0.505	Reference
Yes	71 (3.7%)	6 (2.8%)			1.291 (0.553–3.016)

Significance level, *p* < 0.05; chi-square test and logistic regression analysis were used. Abbreviations: MetS, metabolic syndrome; OR, odds ratio; CI, confidence interval.

**Table 3:** MetS odds ratio according to physical activity and dietary habits.

Variables	Non-MetS (*n* = 1928)	MetS (*n* = 215)	x ^2^	*p*	OR
PA section					
Aerobic PA					
High, 6–7 d/wk	718 (37.2%)	72 (33.5%)	6.584	0.037	Reference
Medium, 3–4 d/wk	664 (34.4%)	60 (27.9%)			1.097 (0.626–3.016)
Low, 0–2 d/wk	546 (28.3%)	83 (38.6%)			1.528 (1.092–2.203)
Strength training					
High, 4–7 d/wk	360 (18.7%)	33 (15.3%)	1.579	0.454	Reference
Medium, 2–3 d/wk	389 (20.2%)	48 (22.3%)			1.336 (0.838–2.130)
Low, 0–1 d/wk	1179 (61.2%)	134 (62.3%)			1.234 (0.828–1.839)
Sedentary time					
Low	833 (43.2%)	69 (32.1%)	15.264	<0.001	Reference
Medium	479 (24.8%)	50 (23.3%)			1.250 (0.853–1.832)
High	616 (32.0%)	96 (44.7%)			1.863 (1.342–2.587)
Dietary section					
Energy intake					
Low	613 (31.8%)	58 (27.0%)	7.337	0.026	1.013 (0.696–1.475)
Medium (recommend)	643 (33.4%)	62 (28.8%)			Reference
High	672 (34.9%)	95 (44.2%)			1.466 (1.046–2.055)
Eating out frequency					
Low, ≤4 times/wk	800 (41.5%)	96 (44.7%)	0.803	0.669	Reference
Medium, 5–6 times/wk	905 (46.9%)	95 (44.2%)			0.866 (0.642–1.169)
High, 1–2 time/day	223 (11.6%)	24 (11.2%)			0.914 (0.570–1.467)
Weekly breakfast frequency					
High, 5–7 days/wk	990 (51.3%)	84 (39.1%)	11.828	0.003	Reference
Medium, 3–4 days/wk	316 (16.4%)	42 (19.5%)			1.562 (1.055–2.314)
Low, 0–2 days/wk	622 (32.3%)	89 (41.4%)			1.706 (1.244–2.339)
Nutrition education					
No	1392 (72.2%)	158 (73.5%)	0.689	0.748	Reference
Yes	536 (27.8%)	57 (26.5%)			1.081 (0.785–1.488)

Significance level, *p* < 0.05; chi-square test and logistic regression analysis were used. Abbreviations: MetS, metabolic syndrome; OR, odds ratio; CI, confidence interval; PA, physical activity.

## Data Availability

Publicly available datasets were analyzed in this study. These data can be found here: [https://knhanes.kdca.go.kr] (accessed on 31 March 2023).
